# Supplementary material for: Cross-Lagged Relationships Between Cognitive Ability and Math Achievement
Source: J Intell. 2025 Oct 31;13(11):138. doi: 10.3390/jintelligence13110138 (PMC12653100; doi:10.3390/jintelligence13110138)
Supplement: Supplementary file 1 [file jintelligence-13-00138-s001.zip › jintelligence-3715616-supplementary.pdf]

Supplementary Material

Covariance Matrix

	KFT2	KFT3	KFT4	D2	D3	D4	KFT1	D1
KFT2	232.120							
KFT3	166.160	213.162						
KFT4	154.033	171.933	202.973					
D2	120.862	122.161	124.872	203.626				
D3	169.299	162.733	174.706	177.577	333.357			
D4	96.758	85.869	80.023	77.174	104.207	143.131		
KFT1	126.467	115.985	99.628	73.916	112.098	71.476	165.413	
D1	167.927	175.713	167.963	155.852	228.572	128.038	121.847	452.527
